# Photons to food: genetic improvement of cereal crop photosynthesis

**DOI:** 10.1093/jxb/eraa077

**Published:** 2020-02-18

**Authors:** Robert T Furbank, Robert Sharwood, Gonzalo M Estavillo, Viridiana Silva-Perez, Anthony G Condon

**Affiliations:** 1 ARC Centre of Excellence for Translational Photosynthesis, Research School of Biology, The Australian National University, Canberra, ACT, Australia; 2 CSIRO Agriculture and Food, Canberra, ACT, Australia; 3 University of Essex, UK

**Keywords:** CO_2_ assimilation, electron transport, grain yield, radiation use efficiency, Rubisco

## Abstract

Photosynthesis has become a major trait of interest for cereal yield improvement as breeders appear to have reached the theoretical genetic limit for harvest index, the mass of grain as a proportion of crop biomass. Yield improvements afforded by the adoption of green revolution dwarfing genes to wheat and rice are becoming exhausted, and improvements in biomass and radiation use efficiency are now sought in these crops. Exploring genetic diversity in photosynthesis is now possible using high-throughput techniques, and low-cost genotyping facilitates discovery of the genetic architecture underlying this variation. Photosynthetic traits have been shown to be highly heritable, and significant variation is present for these traits in available germplasm. This offers hope that breeding for improved photosynthesis and radiation use efficiency in cereal crops is tractable and a useful shorter term adjunct to genetic and genome engineering to boost yield potential.

## Teaser

Photosynthetic performance is a key target for yield improvement in cereal crops. Evidence for genetic variation in photosynthesis is reviewed along with new approaches to measure these traits.

## Introduction: supply, demand, and the challenges ahead

The challenges of sustainably supplying sufficient food to a burgeoning world population, predicted to exceed 9 billion by 2050, have been well documented (FAO 2009; Crist *et al.*, 2017). Apart from the rise in global consumption, we are faced with diminishing arable land through degradation, changes in food preferences as living standards rise across Asia and Africa, and, importantly, the largely negative impacts of climate change and extreme weather events on agricultural production (Dawson *et al.*, 2016). However, focusing on the needs of global agricultural production out to 2050 runs the risk of complacency in our research and crop breeding goals as it is likely that a major imbalance between supply and demand in the global cereal grain market, such as was seen in 2008, will occur long before 30 years have passed (FAO: The state of food insecurity in the world 2011; http://www.fao.org/3/i2330e/i2330e04.pdf). Indeed, global reserves of cereal grain are again low as a proportion of annual global demand and may be precipitously so (http://www.fao.org/worldfoodsituation/csdb/en/).

Recent climate modelling reported by the International Panel on Climate Change (IPCC) is now geographically fine grained enough to allow us to predict not only global increases in average temperature and the impact on agriculture, but also how this maps onto major cereal-producing regions globally (Elbehri *et al.*, 2017; Arneth *et al.*, 2019). Models also predict increases in the frequency of catastrophic weather events and how these will translate into crop losses through heat stress, drought, flood, frost, etc. (Arneth *et al.*, 2019). The impact of climate change on wheat yields from 1990 to 2015 was recently modelled for Australia (Hochman *et al.*, 2017) and found to have been responsible for a 27% decline in yield potential in rainfed environments, despite significant advances in yield resilience made by breeders during this period. If this effect accelerates and is exacerbated by extreme weather events, the current 1% annual improvement in wheat yield potential (Fischer *et al.*, 2014) will not even keep pace with these detrimental effects over the next decade in dry, hot environments, not only in the developing world but also in our major cereal grain-exporting regions. A more than doubling of our annual yield progress is required in the case of all the globally important cereals, and the search for new ‘frontier traits’ to achieve this is now a major breeding focus (Furbank *et al.*, 2019*a*).

## The ‘green revolution’ and improving photosynthesis

**Fig. 1.**
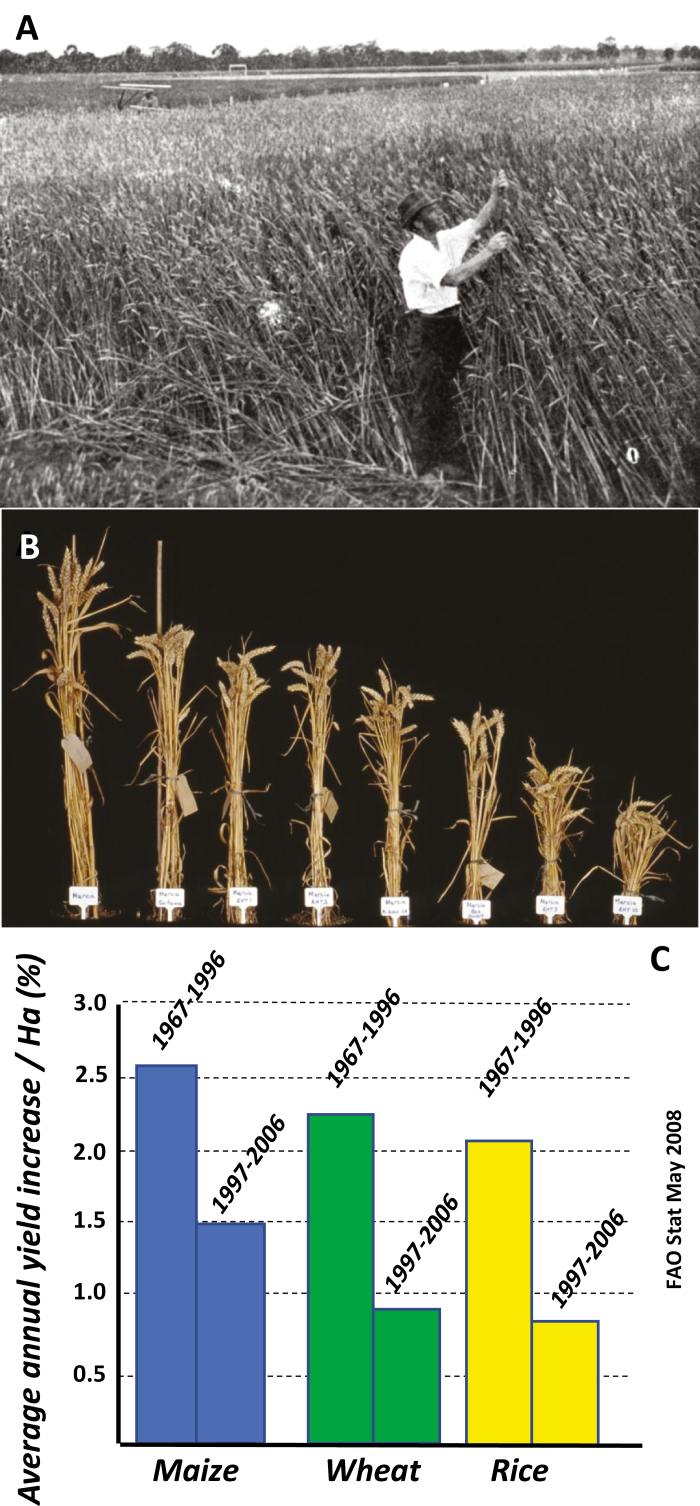
The Green Revolution and global cereal yields. (A) A farmer examining a pre-semi-dwarf wheat crop in 1915 (source: ‘Wheat growing in Australia’ McCarron, Bird & Co, Melbourne, Australia; 1915). (B) Effect of various dwarfing genes on plant stature in near isogenic lines of wheat (sourced from the John Innes Centre image archives). (C) Declining annual yield progress from breeding in the three major cereal crops prior to the 2008 food crisis (FAO, 2009).

The spectacular yield improvements seen in cereals in the 1960s and 1970s and the high annual increases in yield potential in wheat and rice achieved in the subsequent decades has recently declined (Parry *et al.*, 2011; Furbank *et al.*, 2019*a*). It is now widely accepted that the breeding strategies of the green revolution based on improvements in harvest index and grain number and largely driven by adoption of dwarfing genes (Fig. 1) have reached a plateau.

Genetic potential for harvest index in many elite wheat and rice genotypes has reached the theoretical limit of ~0.6 (60% of the plant biomass is harvestable grain; Foulkes *et al.*, 2011). Since the yield equation comprises only harvest index and biomass as factors, there has recently been a major focus on improving wheat and rice biomass without sacrificing harvest index (Parry *et al.*, 2011; Furbank *et al.*, 2019*a*), most readily achievable by improvements in radiation use efficiency (RUE) via increased photosynthetic capacity and efficiency. Since the 2008 food crisis, many hundreds of millions of dollars has now been invested in research to improve photosynthetic performance in model plants and crops, by both transgenic and non-transgenic approaches.

## Candidate gene engineering approaches

Photosynthesis is one of the most intensively studied biochemical processes in plants. Decades of biochemistry and biophysics of mechanisms and processes have been followed by gene suppression work in transgenic plants to ‘titrate’ out levels of key enzymes and determine the limitations to photosynthetic flux afforded by these steps in C_3_ and C_4_ photosynthesis (e.g. Hudson *et al.*, 1992; Furbank *et al.*, 1996; Price *et al.*, 1998; Harrison *et al.*, 2001; von Caemmerer *et al.*, 2005). Modelling has been used extensively to elucidate these limitations to flux under a range of environmental conditions (von Caemmerer, 2000). The contribution of the leaf-level models derived from Farquhar *et al.* (1980) in guiding these experiments has been invaluable in providing a quantitative, mathematical lens with which to examine impacts of gene expression, enzyme activity, and protein level on leaf photosynthetic physiology. Together with systems models (Zhu *et al.*, 2007) and incorporated into field crop simulations (Wu *et al*., 2016, 2019; Yin *et al.*, 2017), modelling continues to guide our strategies for candidate gene selection for manipulation and transgenic plant analysis. Targets for engineering-based approaches have included Rubisco improvement by directed evolution (Wilson *et al.*, 2018), increased expression of Rubisco (Salesse-Smith *et al.*, 2018), reduction of photorespiration by CO_2_-concentrating mechanisms (reviewed in von Caemmerer *et al.*, 2012; Long *et al.*, 2016) and photorespiratory bypasses (South *et al.*, 2019; Shen *et al.*, 2019), overexpression of enzymes important in regeneration of ribulose-1,5-bisphosphate (RuBP) such as sedoheptulose-bisphosphatase (Lefebvre *et al.*, 2005; Driever *et al.*, 2017), modification of photoprotection (Kromdijk *et al.*, 2016), and overexpression of the thylakoid cytochrome *b*_6_*f* complex for ATP production (Simkin *et al.*, 2017; Ermakova *et al.*, 2019*a*). Modification of mesophyll conductance and stomatal conductance to provide better access of CO_2_ to Rubisco has also been explored as an engineering target using proposed CO_2_ porins (reviewed in Groszmann *et al.*, 2017; Condon, 2020).

All these approaches have also been recently reviewed (Simkin *et al.*, 2019) and are the focus of major national and international consortium efforts (e.g. ARC Centre of Excellence for Translational Photosynthesis, https://photosynthesis.org.au/; C_4_ Rice Project, https://c4rice.com/; and Realising Improvements in Photosynthetic Efficiency or RIPE, https://ripe.illinois.edu/). However, while modelling predicts that these modifications alone and in combination could have quantum effects on photosynthetic performance in crops, as yet only a few of these genetically modified traits have been tested in the field in cereals (e.g. Shen *et al.*, 2019) or indeed in any crops apart from tobacco. Recently, interpretation of phenotypes and field performance of transgenic tobacco has also been controversial (Evans, 2019; Fischer *et al.*, 2019). Given the challenges of field translation of genetically modified photosynthetic traits, is there significant genetic variation in photosynthesis in our existing cereal germplasm collections to breed for large increases in biomass and yield? Are these traits heritable and stable and what are the genetics underpinning such variation?

## Genome to phenome: mining allelic variation

**Fig. 2.**
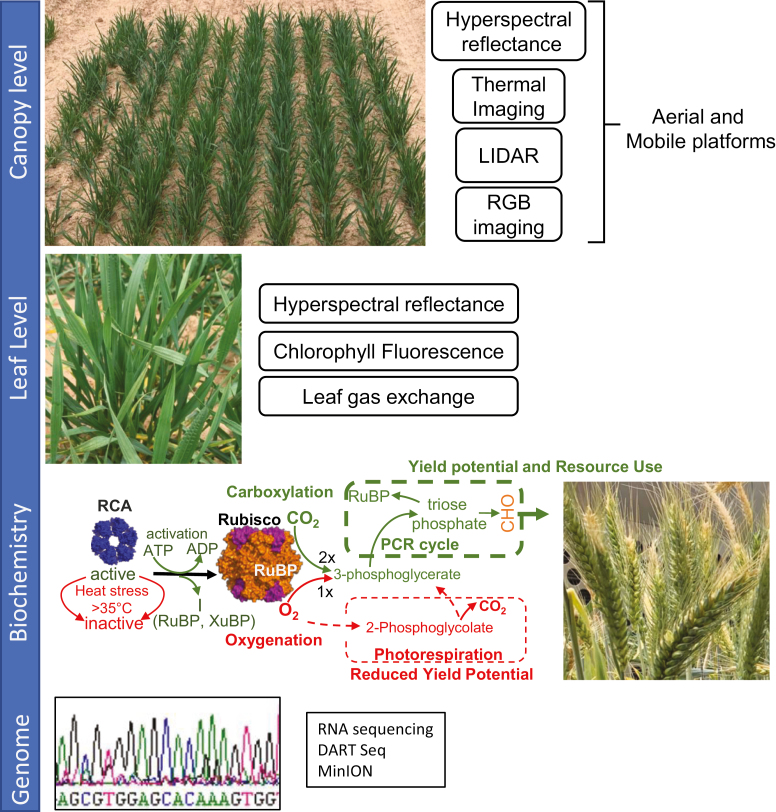
Genome to phenome and back: identification of photosynthetic traits for integration into breeding programmes or gene technologies. Analysis of photosynthetic CO_2_ assimilation from the canopy and leaf level can be achieved through rapid phenotyping techniques (see Furbank *et al*., 2019a, b). These techniques enable rapid determination of photosynthetic parameters that help select germplasm for detailed analyses. At the canopy level, LIDAR is used for non-destructive biomass determination, drones or unmanned aerial vehicles are used for imaging crop canopies which can include RGB cameras for crops coverage, and thermal imaging is used for canopy temperature, which can be utilized for screening germplasm for differences in water use efficiency. At the leaf level, tools such as hyperspectral reflectance can be used to estimate electron transport capacity and *V*_cmax_, in addition to leaf N and leaf mass per area (Silva-Perez *et al.*, 2018). Tools such as MultispecQ (Kuhlgert *et al.*, 2016) and SPAD provide surrogates for leaf N content, with the former measuring electron transport and non-photochemical dissipation of incoming light energy. Determining the underpinning biochemistry and gene sequence diversity is requisite to deploy traits crucial for improving CO_2_ assimilation.

The plummeting costs of genome and transcriptome sequencing means that burgeoning collections of cereal germplasm can be cheaply genotyped, and in many cases high-quality genome re-sequence data are available (Wang *et al.*, 2018; Langridge and Waugh, 2019; Milner *et al.*, 2019). Coupled with the high level of domain knowledge available to identify candidate genes in photosynthetic improvement, this provides an opportunity to mine allelic variation in photosynthetic genes (Furbank *et al*., 2019*a*, b; Fig. 2) and determine their importance. Several initiatives have been established across the globe in wheat, rice, and barley to capture the genetic diversity of both current and historic/heirloom varieties and wild relatives or genome donors in the case of wheat. In wheat, most notable of these are the CIMMYT activities under the Seeds of Discovery program (https://seedsofdiscovery.org/) and the efforts of the Leibniz Institute for Crop Breeding (IPK) to carry out Illumina sequencing of a large proportion of their barley and wheat germplasm collection (https://bridge.ipk-gatersleben.de).

In rice, the International Rice Research Institute (IRRI) have developed a large sequenced diversity panel of *indica* and *japonica* rice genotypes with diverse pedigrees and geographic origins: the 3K population, currently being extended to include 10 000 entries (Wang *et al.*, 2018; http://snp-seek.irri.org/). Maize germplasm collections and genotyping data are perhaps the best developed and have been utilized for many years (reviewed in Romay, 2018). In sorghum, the first C_4_ grass to undergo full genome assembly, genetic resources are also building, with genetic material dating from pre-domestication to current elite lines (Mace *et al.*, 2013; Boyles *et al.*, 2019).

While the genetic resources now exist in a variety of crops to examine allelic variation in all candidate genes/proteins known to be important in controlling photosynthetic flux, Rubisco has been the focus of most efforts to date.

## Exploring the Triticeae tribe for variation in Rubisco catalysis

CO_2_ assimilation within key C_3_ crops is often limited by the catalytic activity of Rubisco (von Caemmerer, 2000; reviewed in Sharwood, 2017). Rubisco is a bifunctional enzyme that can either fix substrate CO_2_ or oxygen to substrate RuBP (Sharwood, 2017). Carboxylation of RuBP is the productive reaction of Rubisco that results in formation of 3-phosphoglycerate that is used for the synthesis of carbohydrate backbones, which are then utilized for plant growth and productive yield, whereas oxygenation is unfavourable because of the production of 2-phosphoglycolate that must be recycled through the photorespiratory pathway (Bauwe *et al.*, 2010). This process consumes energy and releases previously fixed CO_2_ (Sharkey, 1988). Therefore, Rubisco is regarded as an inefficient catalyst, with a catalytic cycle of 2–3 carboxylations per second, a meagre catalytic efficiency, and poor specificity for CO_2_ (Sharwood, 2017). In addition, Rubisco requires the assistance of Rubisco activase (RCA) that is required to maintain and modulate enzymatic activity through metabolic repair of the activity that removes inhibitory sugar phosphates (Mueller-Cajar, 2017). These include the misfiring product xylulose bisphosphate (XUBP), the nocturnal inhibitor carboxyarabinitol-1-phosphate (CA1P), and substrate (Parry *et al.*, 2008). Surprisingly, RCA is thermolabile within C_3_ crops, with activity dropping significantly as temperatures exceed 35 °C (Crafts-Brandner and Salvucci, 2000). To circumvent Rubisco catalytic inefficiencies, terrestrial plants devote significant amounts of leaf nitrogen (N) to synthesize large amounts of Rubisco to ensure appropriate CO_2_ assimilation. However, this has burdened agriculture with the high use of N fertilizers to ensure sufficient Rubisco is synthesized for assimilating carbon for growth and yield. Therefore, Rubisco and RCA are two primary targets for improving CO_2_ assimilation by ameliorating catalysis to improve both photosynthetic N use efficiency and photosynthetic water use efficiency.

**Fig. 3.**
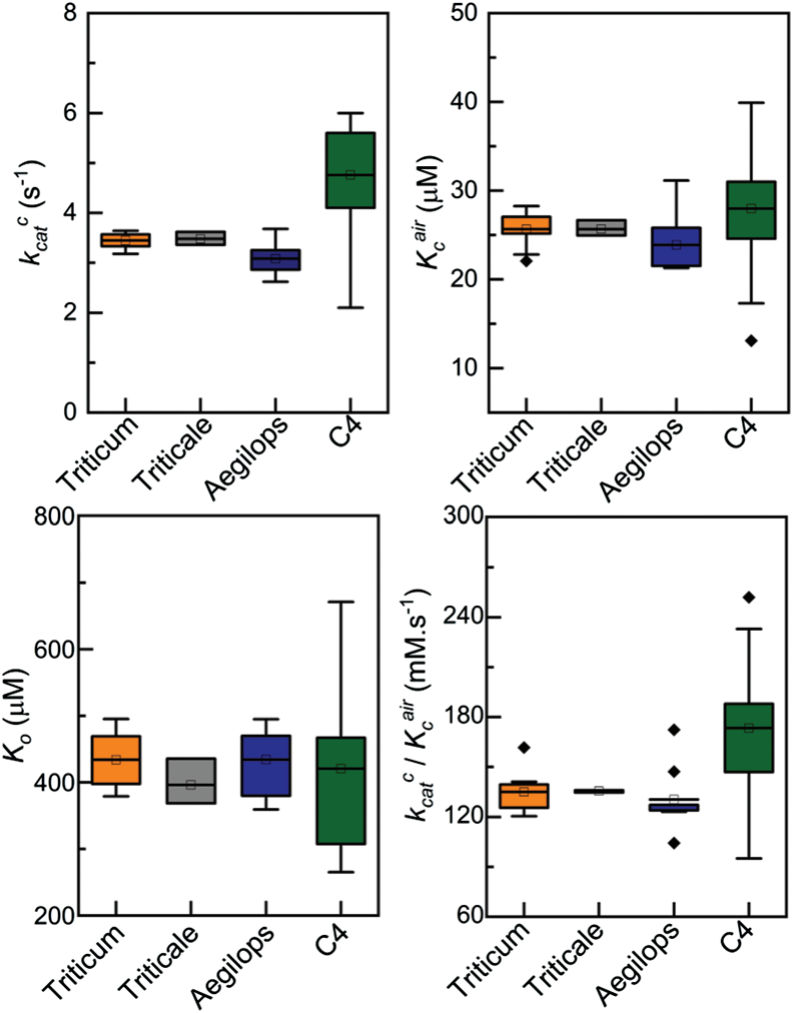
Exploring Rubisco catalytic diversity within Triticeae and C_4_ plants. Variation in key Rubisco catalytic parameters from Triticeae and compared with plant C_4_ Rubisco. Parameters include the carboxylation speed, *k*_cat_^c^, the Michaelis constants for CO_2_ and O_2_ and carboxylation efficiency (*k*_cat_^c^/*K*_c_^air^). Rubisco catalytic data for the Triticeae tribe were replotted from Prins *et al.* (2016) into box plots alongside C_4_ Rubisco. *K*_c_^air^ was calculated for Triticeae Rubisco using the formula *K*_c_^air^ (μM)=*K*_c_(1+O/*K*_o_), where *K*_c_ and *K*_o_ are the Michaelis constants for CO_2_ and oxygen, respectively, and O is the atmospheric O_2_ concentration—252 μM. The C_4_ Rubisco parameters were replotted from Jordan and Ogren (1983); Kubien *et al*. ( 2008); Whitney *et al.* (2011); and Sharwood *et al*. (2016a, b). In each box plot, the black square and the horizontal bar indicate the mean. The lower and upper edge of each box indicate the interquartile (25–75%) range of the values reported. The whiskers extend to 1.5 times the interquartile range.

Screens of *in vitro* Rubisco catalytic parameters among terrestrial plants (C_3_ and C_4_) and algae have revealed substantial diversity in Rubisco catalysis and identified versions better suited to current and predicted future climate scenarios (Galmés *et al*., 2014; Orr *et al.*, 2016; Sharwood *et al.*, 2016*a*; Young *et al.*, 2016; Heureux *et al.*, 2017). Three catalytic parameters are required for modelling to assess performance. These are: (i) the Michaelis constant for CO_2_ in air (*K*_c_^air^); (ii) carboxylation speed (*k*_cat_^c^); and (iii) the specificity for CO_2_ as opposed to O_2_ (*S*_c/o_) (Sharwood, 2017). Until recently, little information was known about the diversity of Rubisco catalysis within the Triticeae tribe. Analysis of 25 species with the tribe demonstrated diversity of Rubisco catalysis, with variation observed in *k*_cat_^c^, *K*_c_^air^, and *K*_o_ (the Michaelis constant for oxygen) (Fig. 3A–C; (Prins *et al.*, 2016). Interestingly, Triticum species showed improved carboxylation efficiency at 21% O_2_ compared with Aegilops relatives (Fig. 3D; Prins *et al.* (2016). Understanding the source of this change, probably variation in the sequence of Rubisco small subunits, will provide key information to further improve wheat Rubisco catalysis.

Further exploration of the Triticeae tribe is required to fully assess diversity in Rubisco catalysis. Identifying catalytic switches in the Rubisco large and small subunits will open up new opportunities for improving wheat CO_2_ assimilation. However, it is evident in Fig. 3 that C_4_ Rubisco provides solutions to improve the current wheat forms. The *k*_cat_^c^ and carboxylation efficiency (*k*_cat_^c^/*K*_c_^air^) of C_4_ Rubisco outperform those of wheat. Comparison of Rubisco specificity is difficult as Prins *et al.* (2016) used a different technique for measuring *S*_c/o_ from that for C_4_ plants presented. Nevertheless, it is evident that maize Rubisco provides improved CO_2_ assimilation when compared at 25 °C (Sharwood *et al.*, 2016*b*).

While substantial catalytic diversity exists within the Rubisco superfamily of enzymes, more interrogation of catalytic diversity is required. Efforts to include Rubisco as a breeding target have been limited due to the labour-intensive measurements of Rubisco catalysis. High-throughput surrogates for Rubisco capacity and catalytic properties measured on intact leaves offer hope of tractable screening methods, and these are discussed in the following sections.

## Genetic diversity in cereal crop photosynthesis

High-throughput direct measurement of carbon assimilation presents a large technical challenge, even using so-called ‘rapid’ gas analysis techniques (e.g. Stinziano *et al.*, 2017). Exploring genetic diversity for photosynthetic traits in germplasm collections and large structured mapping populations of cereals has long been considered a laborious and almost an intractable task (Parry *et al.*, 2011; Furbank *et al*., 2019*a*, b). Single point measurements of assimilation rate on flag leaves of spring and winter wheat have been seen to have both a positive and negative correlation with yield and year of release (Murthy and Singh, 1979; Evans, 1993; Reynolds *et al*., 1994, 2000; Fischer *et al.*, 1998; Fischer *et al.*, 2010; Sadras *et al.*, 2012; Gaju *et al.*, 2016; Tang *et al.*, 2017). There is of course the complication in such studies of which leaf and at which developmental stage to measure and how to compare germplasm with vastly different phenology (Parry *et al.*, 2011). Obtaining gas exchange data in the field is slow, with stomatal limitation often complicating the measurement (Condon *et al.*, 2004; Feng *et al.*, 2018). While even more time-consuming than assimilation measured at ambient CO_2_, CO_2_ response curves or ‘*A* versus *C*_i_’ curves potentially provide a better estimate of photosynthetic traits as they allow extraction of estimates of Rubisco amount and kinetic efficiency (*V*_cmax_) and electron transport capacity (*J*) using the modelling frameworks discussed above (Farquhar *et al.*, 1980; Silva-Perez *et al.*, 2018, 2019). Given the time constraints for measurement, a limited number of studies have been done with diversity panels of wheat using gas exchange, with few of these done in the field or incorporating leaf structural traits.

**Fig. 4.**
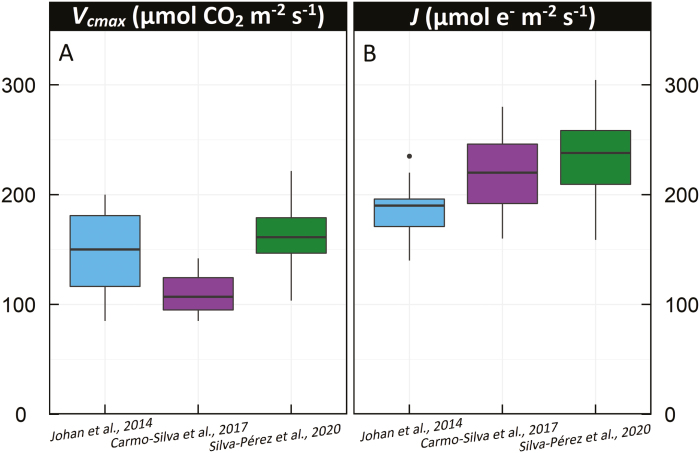
Genetic variation in wheat for (A) Rubisco activity (*V*_cmax_) and (B) electron transport rate (*J*). *V*_cmax_ and *J* for 11 wheat genotypes measured in young plants grown in a controlled-environment growth cabinet (Jahan *et al.*, 2014), 64 winter wheat genotypes grown in the field in the UK (Carmo-Silva *et al.*, 2017), and 74 spring wheat genotypes measured in a glasshouse (only high N treatment shown) and field in Australia and Mexico (Silva-Perez *et al.*, 2018, 2020).

Figure 4 summarizes the most recent of these published data (Driever *et al.*, 2014; Jahan *et al.*, 2014; Carmo-Silva *et al.*, 2017; Silva-Perez *et al.*, 2018, 2019). Across elite germplasm (including historic germplasm sets), there appears to be substantial genetic variation in the modelled parameters reflecting Rubisco capacity (*V*_cmax_) and photosynthetic electron transport (*J*) (Fig. 4). In the larger data sets of Silva-Pérez *et al*. (2020) and Carmo-Silva *et al.* (2017), genetic variation in *J* appeared to be larger than in *V*_cmax_, but this was not the case in the smaller germplasm set of Jahan *et al.* (2014). Heritability of these gas exchange-derived traits can be quite high (broad-sense heritabilities of 0.31–0.76 have been reported for *A* in winter wheat grown in the UK; Carmo-Silva *et al.*, 2017) and for spring wheats grown in Mexico and Australia, heritability of modelled parameters *V*_cmax_ and *J* were also as high as 0.7 (Silva-Pérez *et al*., 2020). These data suggest that photosynthetic traits are genetically robust enough to breed with and that substantial variation is present in elite material and potentially even greater variation in landraces and wild relatives.

In rice, several studies have been carried out on diverse germplasm to investigate genetic variation in *A* from gas exchange. In a collection of 20 diverse *japonica* and *indica* genotypes, light-saturated *A* at ambient CO_2_ concentration varied by >40% from lowest to highest genotypes (Jahn *et al.*, 2011). However, low heritability of *A* observed in this study (0.17) is indicative of the challenges in using a single point assimilate rate to find the genetic architecture underlying photosynthetic traits.

## High-throughput phenomics: accelerating germplasm screening from organs to canopy

### Hyperspectral reflectance

As discussed above, high-throughput surrogates for photosynthetic traits traditionally derived from gas exchange are a priority because: (i) the large scale of experiments necessary to screen germplasm diversity sets and mapping populations proves too costly; (ii) time of day and seasonal variation affect trait expression, compressing time available for measurements; and (iii) measurements may need to be made on several leaves/plant organs at different developmental stages in the crop life cycle to obtain a comprehensive analysis. To obtain accurate mapping of photosynthetic traits in a structured population, such as a recombinant inbred set with high-density genetic maps usually requires in excess of 150 lines grown with replication, in the field, preferably across multiple seasons and often multiple locations (Collard *et al.*, 2005). Given that leaf-level measurements can require between two and six replicates per field plot, even at a single developmental stage, this would mean that with 3-fold replication at both the genotype and technical level. a minimum of 1350 individual measurements must be made. If multiple leaf classes at several stages of development are added to the experiment, the phenotyping rapidly becomes unachievable in a reasonable amount of time, particularly if time of day effects are to be avoided. For genome-wide association panels, the size of the experiment may increase to 1000 germplasm entries if gene-level resolution is desired (Ingvarsson and Street, 2011), exacerbating the problem.

Recent advances in machine learning coupled with optical sensing systems at the leaf and canopy level offer a potential solution to this issue. Leaf and canopy spectral reflectance measurements can now be made using affordable visible/near infrared spectrometers or more costly full range visible, NIR, SWIR spectrometers, enabling the collection of many hundreds of wavelengths from below 400 nm to 2500 nm. Predictions of leaf traits from spectral reflectance data are made using machine learning to generate statistical models between every wavelength of reflected light from leaves and the trait of interest measured with traditional methods (termed a ‘training set’). These models are then either validated on another set of germplasm or the training set is divided into a training and validation set for testing (Silva-Perez *et al.*, 2018).

This approach has resulted in prediction of leaf traits related to photosynthesis such as leaf N, phosphorus, mass per area, and photosynthetic traits such as *A*, *J*, and *V*_cmax_ in plants ranging from trees to annual C_3_ and C_4_ crops (e.g. Serbin *et al.*, 2012; Ainsworth *et al.*, 2014; Singh *et al.*, 2015; Yendrek *et al.*, 2017; Silva-Perez *et al.*, 2018). This technique has recently been extended to predictions of respiration rate in wheat (Coast *et al.*, 2019) and field evaluation of transgenic plants with altered photosynthesis (Meacham-Hensold *et al.*, 2019). The attraction of hyperspectral reflectance models is that the collection of spectra can take <20 s per leaf and does not require any equilibration of the leaf in the sensor. There are, however, obstacles to the widespread use of a machine learning to examine genetic variation in crop photosynthetic traits as acquiring the data necessary to build a training set requires many hundreds or even thousands of measurements using the older, slower traditional methods. If training sets are not sufficiently large, containing a wide range of germplasm and even different leaf classes/developmental stages and environments, spurious ‘overfitting’ occurs and the predictive power of models is diminished for leaves which fall outside the panel used to generate the model (see Heckmann *et al.*, 2017; Coast *et al.*, 2019).

### Chlorophyll fluorescence

While direct measurement of carbon fixation in high throughput is problematic, estimation of photosynthetic electron transport capacity and related leaf-level efficiencies can be tractable in high throughput using chlorophyll fluorescence techniques without the use of models or proxies (reviewed in Maxwell and Johnson, 2000; Murchie and Lawson, 2013). Compact, commercial pulse amplitude-modulated chlorophyll fluorescence (PAM) systems are available (Cessna *et al.*, 2010; Kuhlgert *et al.*, 2016) which apply a brief saturating flash of light via a high-intensity LED, in the light or dark, allowing calculation of either the photosynthetic electron transport rate (ETR or *+*) or the intrinsic light-harvesting efficiency of PSII (dark-adapted *F*_v_/*F*_m_). NPQ (non-photochemical quenching, resulting primarily from dissipation of energy as heat) can also be calculated (Maxwell and Johnson, 2000).

Deploying PAM at canopy level in a field crop presents difficulties in both obtaining a uniform saturating flash and in interpreting data from the complex 3D structure of the canopy. For canopy-level measurements, non-imaging fluorescence sensors or light-induced fluorescence transients (LIFTs) which apply a series of ‘flashlets’ to a spot of the canopy up to a few centimetres wide may be useful for field phenotyping (Keller *et al.*, 2019), or imaging sensors for sun-induced fluorescence (SIF) mounted on drones, manned aircraft, or even satellites may be used to generate high-throughput phenotyping data (Zhang *et al.*, 2018; Camino *et al.*, 2019; Furbank *et al.*, 2019b).

Aerial and satellite remote sensing using chlorophyll fluorescence has so far been utilized mainly to study natural vegetation and ecosystems or for horticulture with tree species (Furbank *et al.*, 2019b) and is only recently being used for exploring photosynthetic variation in crops (Camino *et al.*, 2019).

In contrast, leaf-level chlorophyll fluorescence has been used to explore diversity in chloroplast electron transport and, to a limited degree, the genetics behind this variation

Wheat cultivars more tolerant to heat stress have been identified based on higher *F*_v_/*F*_m_ from dark-adapted, detached leaves from a diversity panel of 41 lines of different origins (Sharma *et al.*, 2015). Although commercial ‘Imaging PAM’ systems and hand-held devices are available for the estimation of chlorophyll-based parameters, they have been costly and have had limited application to high-throughput field phenotyping.

The development of new microelectronics and sensors has led to the design of hand-held instruments amenable for larger screenings. For example, the MultisepQ sensor can rapidly measure several fluorescence-based photosynthetic parameters and other physical leaf traits in field situations at a low cost (Kuhlgert *et al.*, 2016), such as SPAD, linear electron flow (LEF), and PSII quantum yields (Kuhlgert *et al.*, 2016). The MultispeQ sensor has been used successfully to assess the effects of abiotic stresses (i.e. heat and drought) in *Phaseolus* (Traub *et al.*, 2018) and cowpea (Mwale *et al.*, 2017), and characterization of photosynthetic traits in potato (Prinzenberg *et al.*, 2018) and transgenic tobacco plants with alternative electron transport (Gómez *et al.*, 2018). This system also features an open platform for metadata annotation, robust data acquisition, and easy sharing of information.

To investigate the utility of the MultispeQ sensor in a cereal crop, we screened a subset of the pre-breeding ‘Vavilov’ wheat population representing the genetic diversity of the panel (Riaz *et al.*, 2017) and 10 commercial wheat varieties. A 4-fold difference between the highest and lowest LEF values was detected (GME *et al*., unpublished). Both Vavilov and commercial checks spread over a similar range of LEF, between 20 µmol e m^–2^ s^–1^ and 80 µmol e m^–2^ s^–1^. The mean LEF for Vavilov was slightly higher and there were few lines with higher LEF than commercial wheats. In addition, concurrent measurements of SPAD with the same sensor demonstrated a dynamic response of LEF to N content mostly in the Vavilov lines, with commercial varieties presenting higher N. High-throughput screening for leaf photosynthetic-related traits using sensors such as MultispeQ can be useful to identify new diversity in photosynthetic capacity and efficiency in breeding programmes or during surveys of diversity panels at relatively low cost.

Photosynthesis by wheat spikes can provide up to 25% of total grain carbohydrate during grain filling, and this contribution could be higher under stressful conditions. It is anticipated that genetic variability for the spike photosynthesis trait exists, but phenotyping this photosynthetic capacity in this organ is challenging due to their complex structure and to the unknown capacity for re-fixation of carbon respired from the developing grain. The latter makes traditional gas exchange difficult to interpret because it measures net CO_2_ uptake by the spike. Moreover, and although gas exchange chambers for 3D organs such as spikes may be custom-built (Sanchez-Bragado *et al.*, 2014; Fortineau and Bancal, 2018), screening of individual wheat spike photosynthesis by gas exchange in the field is not practical for high-throughput phenotyping

Imaging of chlorophyll fluorescence using PAM of intact or detached ears offers an alternative to estimate photosynthetic capacity of wheat spikes. The contribution of photosynthesis and other energy dissipation processes to ETR (a surrogate for photosynthetic capacity) can be monitored by measuring the quenching of chlorophyll fluorescence over a range of light intensities. A mathematical model (von Caemmerer, 2000) can then be fitted to the ‘light response curve’ of ETR to calculate Φ, the efficiency of light capture; θ, qualitative ‘curvature factor’; and *J*_max_, maximum potential electron transport rate. *J*_max_ is widely used as a measure of leaf ‘light use capacity’ analogous to the modelled parameter *V*_cmax_ as a metric of ‘Rubisco capacity’ (Fig. 6).

**Fig. 5.**
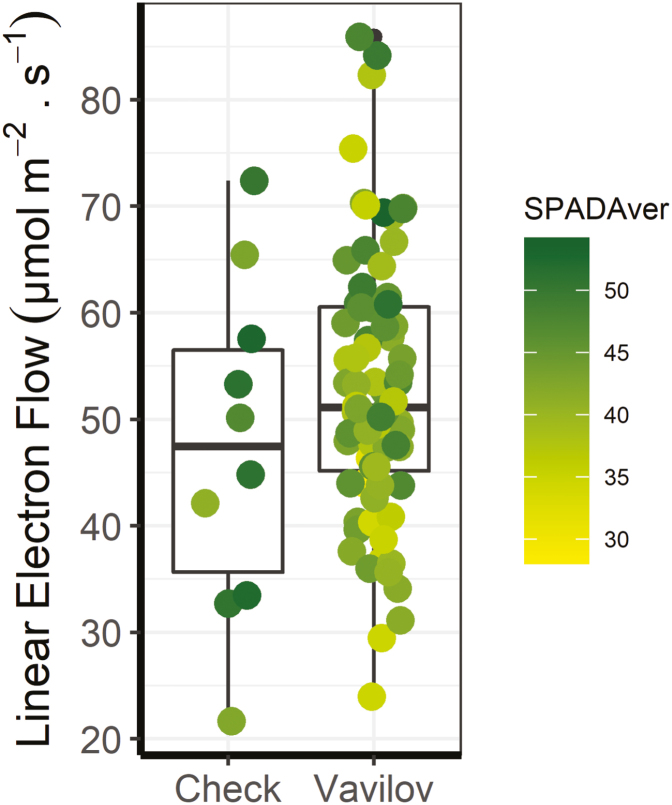
Rapid screening of linear electron flow (LEF) in wheat using chlorophyll fluorescence. Measurements of LEF were taken with three different MultispeQ sensors (Kuhlgert *et al.*, 2016) between 09.00 h and 14.00 h on the same, youngest, fully expanded leaf on a subset (76 lines) of the Vavilov collection and 10 commercial wheat varieties. The box plot shows the spread of LEF values in both sets while the individual points represent the averages for individual lines coloured by the average of the SPAD values simultaneously measured with MultispeQ. In each box plot, the black square and the horizontal bar indicate the mean. The lower and upper edge of each box indicate the interquartile (25–75%) range of the values reported. The whiskers extend to 1.5 times the interquartile range.

**Fig. 6.**
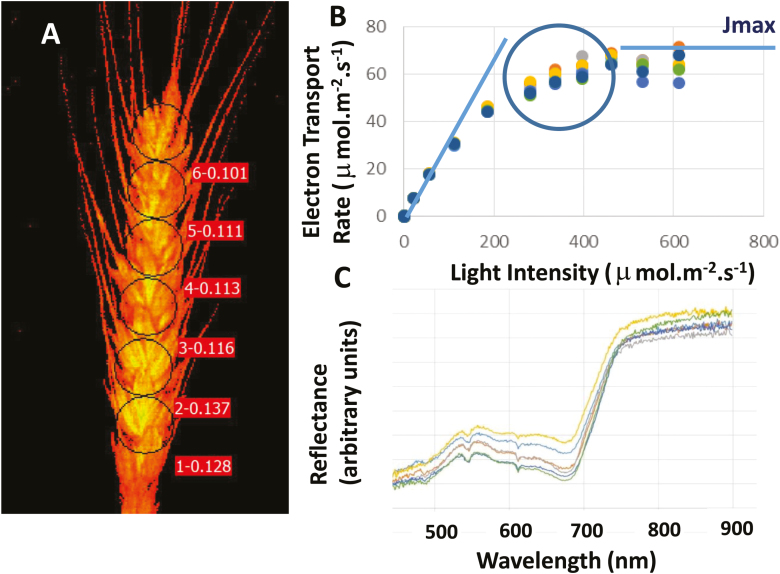
Hyperspectral reflectance imaging and machine learning for predicting photosynthetic traits in wheat spikes. (A) PAM maximum fluorescence image of a wheat spike showing six regions of interest. The three parameters which can be extracted from a model of the response of ETR to light intensity are: Φ, efficiency of light capture; *J*_max_, the electron transport ‘capacity’; and θ, a qualitative ‘curvature factor’. ETR is calculated from chlorophyll fluorescence images. (B) Typical light–response curve for individual regions of interest. (C) Average reflectance spectra from hyperspectral imaging for the six regions in (A) and (B).

However, this technique based on a commercially available PAM fluorometer is not scalable to proximal remote-sensing buggies or to drones, and thus not applicable to canopy-based measurements in the field. Alternatively, hyperspectral imaging sensors mounted on movable platforms would be scalable to field-based application. Machine learning algorithms can deconvolute the hyperspectral signal from wheat leaves to estimate photosynthetic parameters obtained by gas exchange measurements (e.g. Silva-Perez *et al.*, 2018). Similarly, training a statistical model for hyperspectral prediction of wheat spike ETR derived from PAM could be possible. For example, the light response curve of measured ETR with Imaging PAM could be used as a training set and combined with reflectance spectra from hyperspectral imaging of spikes to build predictive models for all key chlorophyll fluorescence parameters (*J*_max_, θ, and Φ) using statistical approaches such as partial least squares regression, random forest, a support vector machine, or neural networks. The combination of hyperspectral imaging and existing methods to detect spikes using image analyses with deep learning (Hasan *et al.*, 2018) or neural networks (Qiongyan *et al.*, 2017) could be instrumental in identifying genetic diversity in wheat spike photosynthesis.

## Phenome to genome: QTLs and the genetic architecture of photosynthesis traits

Establishing allelic variation in genes known to encode important proteins in photosynthesis (genome to phenome) relies upon pre-existing knowledge of these candidates (Fig. 2). Using high-throughput phenomics to generate trait associations with genomic regions can identify completely unknown genes affecting photosynthetic performance. Understanding the genetic architecture of photosynthetic performance can identify quantitative trait loci (QTLs) or markers which are useful breeding tools for marker-assisted selection or, if fine grain genetic maps and re-sequence data are available, single nucleotide polymorphisms (SNPs) can be identified in candidate genes which can be used to understand genetic and biochemical mechanisms and drive gene editing and transgenic approaches.

Despite promising data on genetic diversity and heritability of photosynthetic traits, there are surprisingly few examples of QTL mapping of these parameters in wheat, given its importance globally. There have, however, been several studies targeted to photosynthesis-related traits such as stress tolerance, canopy temperature, stomatal conductance, and transpiration efficiency (e.g. Mason *et al.*, 2013; Rebetzke *et al.*, 2013). Recently, Molero *et al.* (2019) explored the genetic basis of biomass accumulation and RUE in wheat by a genome-wide association study (GWAS). A panel of 150 elite spring wheat genotypes including many landrace and synthetically derived lines were examined using more traditional approaches such as measuring yield components and biomass accumulation over time combined with estimated intercepted radiation. Marker–trait association identified 94 SNPs significantly associated with yield, agronomic, and phenology-related traits along with RUE and final biomass (BM_PM) at various growth stages that explained 7–17% of phenotypic variation. Common SNP markers were identified for grain yield, BM_PM, and RUE on chromosomes 5A and 7A. While the density of the genetic map was not sufficient to fine-map and identify single candidate genes, several QTLs encompassed genes involved in processes associated with photosynthesis such as reactive oxygen detoxification and photoprotection of PSII.

In rice, several QTL studies have been carried out mapping measurements of *A* with QTLs found on chromosomes 3, 4, 5, 6, 8, and 11 (Teng *et al.*, 2004; Adachi *et al*., 2011, 2019; Gu *et al.*, 2012). Many of these QTLs account for only a small proportion of variation or are dependent on genetic background (Adachi *et al.*, 2019). Consequently, only a small number of these loci have been fine-mapped and causative genic SNPs identified which underpin genetic variation for *A* in rice. Most notably, GREEN FOR PHOTOSYNTHESIS, originally thought to be associated with Rubisco carboxylation efficiency, has now found to be a determinant of leaf thickness, chlorophyll content, and canopy chlorophyll distribution (Takai *et al.*, 2013; Hirotsu *et al.*, 2017), and Car8, a transcription factor affecting photosynthetic capacity (Adachi *et al.*, 2017), is also involved in control of flowering time and duration. The complex interactions between flowering time, phenology, tillering, N partitioning, and photosynthetic traits add to the difficulty of finding robust trait associations, and sophisticated statistical treatments such as deep learning (Zou *et al.*, 2019) may be necessary to tease apart these confounding factors.

## Photosynthetic capacity and leaf nitrogen content

Silva-Pérez *et al*. (2020), in a study exploring genetic diversity in wheat for photosynthetic traits, introduce the concept of photosynthetic capacity (*P*_c_) and photosynthetic efficiency (*P*_eff_, i.e. *V*_cmax_, or *J* per unit leaf N) to describe the drivers of variation in modelled leaf-level traits. It has been widely reported that in cereal crops Rubisco amount and activity are closely related to N nutrition and leaf N content per unit area (Evans, 1983, 1989; reviewed in Evans and Clarke, 2019). It has also been reported that a reduction in flag leaf size, associated with the introduction of the *Rht* dwarfing genes to green revolution wheats, increased N and photosynthetic capacity on a leaf area basis in subsequent varieties (Bishop and Bugbee, 1998). Leaf N content is also important in cereals, as a large proportion of grain protein is derived from leaf protein remobilized during senescence (see Evans and Clarke, 2019). There is concern that improvements in photosynthetic capacity may require increased agronomic N fertilizer use over and above that already required to realize the gains of the green revolution genotypes (Parry *et al.*, 2011).

**Fig. 7.**
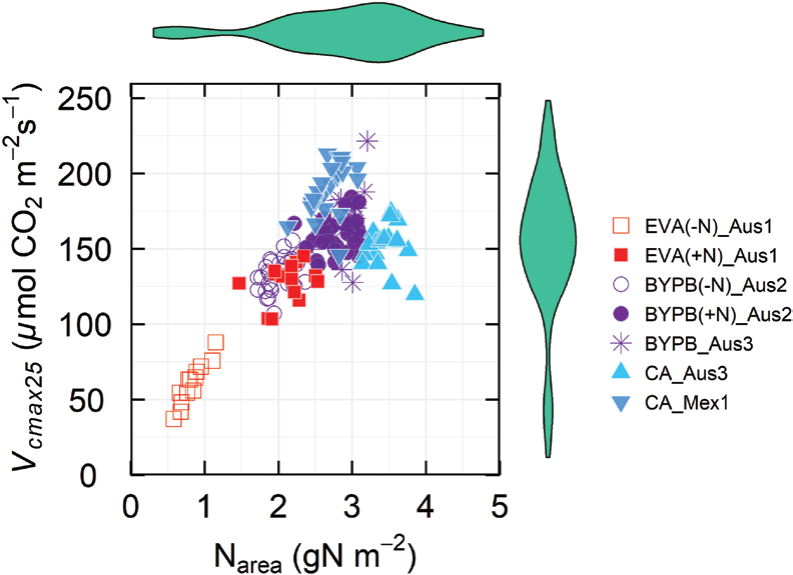
Diversity of Rubisco content (*V*_cmax25_) per unit of nitrogen per leaf area in 74 spring wheat genotypes measured in different environments and growing stages. EV and BYP are two different collections of wheat genotypes grown in glasshouse conditions with two nitrogen levels (–N, +N). BYP and CA are two different wheat collections of wheat genotypes grown in the field in Australia (Aus3) and in Mexico (Mex1). See Silva-Pérez *et al*., 2020) for experimental details. In the *x* and *y* margins, a violin plot is shown representing the distribution of N_area_ and *V*_cmax_, respectively. EV, early vigour set; BYP, high yielding set of wheat genotypes in Australia. (C) High yielding set of wheat genotypes from CIMMYT measured after anthesis. A and B designations at the end of the genotype acronyms refer to the plant growing stages at the time of the measurements, A, after anthesis, B, before anthesis.

Figure 7 shows the relationship between leaf N and *V*_cmax_ on an area basis in diverse wheat genotypes (data from Silva-Pérez *et al*., 2020). Clearly, the amount and activity of Rubisco are related to leaf N, particularly when N supply is restricted (open squares in Fig. 3), but, at higher leaf N values more relevant to agronomic N application levels, a wide range of *V*_cmax_ values were obtained across the genotypes tested. Similar results are also found for *J* (Silva-Pérez *et al*., 2020). Given the high heritability of these modelled parameters in wheat (Silva-Pérez *et al*., 2020 and references therein), either the catalytic efficiency of Rubisco or the electron transport efficiency is superior in some wheat genotypes or the partitioning of leaf N to these protein components in leaves is different between genotypes. These two options cannot be separated with the data available but, clearly, photosynthetic capacity per unit leaf N (*P*_eff_) has a strong genetic component and there is considerable variation in wheat. Given the robust models available to predict these parameters from leaf hyperspectral reflectance data (Silva-Perez *et al.*, 2018), it should be possible to screen large diversity sets and mapping populations to understand and exploit the underlying genetic control, and this work is underway in the IWYP consortium (see https://iwyp.org/funded-projects/).

## Source/sink regulation: feedback or feedforward?

It has frequently been pointed out that improvements in photosynthetic performance will only be translated into improved grain yield if ‘partitioning’ between source and sink and associated signalling processes are accounted for (reviewed in Reynolds *et al.*, 2012). Indeed, it has long been a point of conjecture among crop physiologists as to whether cereal yields are source or sink limited (Parry *et al.*, 2011; Reynolds *et al.*, 2012). Experiments with free air CO_2_ enrichment of a variety of crop species, while often showing increases in yield due to elevated photosynthetic activity, also frequently show sink strength-mediated feedback limitation and limited impact on yield, thought to be due to negative effects of sugar signalling on photosynthetic gene expression, particularly Rubisco and leaf N levels (Ainsworth and Long, 2005). Various reports over the last 20 years have indicated strong feedback links between sink and source capacity, but the mechanisms for this signalling are complex (Wingler, 2018; Paul *et al.*, 2018). While sucrose itself is something of a weak signal for sugar feedback on leaf processes, recent evidence suggests that the signalling metabolite trehalose-6-phosphate (T6P) may perform this role and also affect phloem transport processes via regulation of the SWEET membrane transporters (Paul *et al.*, 2018).

While sugar feedback could be an impediment to realization of increased yield from photosynthetic improvement, there is also evidence of feedforward effects of photosynthetic performance on sink development. In wheat, the final size of the ear and floret number is determined early in development when the inflorescence is still within the sheath (Reynolds *et al.*, 2009). Supply of photoassimilate at this stage and at flowering can strongly influence floret number, floret fertility, and final grain number (Fischer, 1985; Reynolds *et al.*, 2005; Broberg *et al.*, 2019). The coordination between sink and source capacity at this developmental stage may be why it is difficult to separate the contribution of photosynthesis from sink strength when examining the basis for historic yield improvement (see Furbank *et al.*, 2019a). Such coordination may also provide hope that increased photosynthetic capacity and efficiency could actually translate to improved yield by feedforward effects on sink capacity.

## Translation of photosynthetic performance to yield and resilience on farm

Current large investments in research for improved crop photosynthetic performance have varying timelines for delivery of new cereal varieties to farmers. Improved tools for phenotyping photosynthesis could identify material for crossing which can then be utilized in breeding programmes almost immediately, but understanding the genetic architecture of photosynthesis, and finding QTLs and causative SNPs in new gene targets requires appropriate diversity sets or biparental populations and then creation of near isogenic lines or gene-edited ‘allele mimics’ to demonstrate causality. This may reasonably be expected to take 3–5 years for the results of field-based activities to be available to crop breeders, assuming genetic material is in place. Deployment of genetically modified traits, once prototyped in model systems, may take even longer after careful validation in multiple environments and in multiple transgenic events. Complex multigene pathway engineering, while becoming rapidly more tractable with synthetic biology and gene editing (for C_4_ rice, see Ermakova *et al.*, 2019b), would require 10 years plus research and then pre-breeding before delivery to breeders as a trait. While a population of 10 billion people on earth seems some way off, there is definitely no time to waste.
